# Cell resistance to the Cytolethal Distending Toxin involves an association of DNA repair mechanisms

**DOI:** 10.1038/srep36022

**Published:** 2016-10-24

**Authors:** Elisabeth Bezine, Yann Malaisé, Aurore Loeuillet, Marianne Chevalier, Elisa Boutet-Robinet, Bernard Salles, Gladys Mirey, Julien Vignard

**Affiliations:** 1 INRA, UMR1331, Toxalim, Research Center in Food Toxicology, F-31027 Toulouse, France; 2 Université de Toulouse, Institut National Polytechnique de Toulouse, F-31030 Toulouse, France; 3 Université de Toulouse, Université Paul Sabatier, F-31062 Toulouse, France

## Abstract

The Cytolethal Distending Toxin (CDT), produced by many bacteria, has been associated with various diseases including cancer. CDT induces DNA double-strand breaks (DSBs), leading to cell death or mutagenesis if misrepaired. At low doses of CDT, other DNA lesions precede replication-dependent DSB formation, implying that non-DSB repair mechanisms may contribute to CDT cell resistance. To address this question, we developed a proliferation assay using human cell lines specifically depleted in each of the main DNA repair pathways. Here, we validate the involvement of the two major DSB repair mechanisms, Homologous Recombination and Non Homologous End Joining, in the management of CDT-induced lesions. We show that impairment of single-strand break repair (SSBR), but not nucleotide excision repair, sensitizes cells to CDT, and we explore the interplay of SSBR with the DSB repair mechanisms. Finally, we document the role of the replicative stress response and demonstrate the involvement of the Fanconi Anemia repair pathway in response to CDT. In conclusion, our work indicates that cellular survival to CDT-induced DNA damage involves different repair pathways, in particular SSBR. This reinforces a model where CDT-related genotoxicity primarily involves SSBs rather than DSBs, underlining the importance of cell proliferation during CDT intoxication and pathogenicity.

The Cytolethal Distending Toxin (CDT) is a virulence factor produced by many pathogenic bacteria^1^. CDT is a tripartite holotoxin generally composed of two regulatory subunits (CdtA and CdtC) and one catalytic subunit (CdtB)^2^. As an exception, CdtB from the typhoid toxin, identified in *Salmonella enterica* serovar Typhi, is associated with another catalytic subunit (PltA) and regulatory subunits (PltB)^3^. Sequences and structures of the different CdtB subunits are highly conserved^4^ and the CdtB virulence properties have been documented in many cases^5^,^6^. Indeed, mice infected with *Helicobacter hepaticus* developed hepatic dysplasic nodules, whereas mice infected with the CdtB-deficient strain did not^5^. Moreover, many of the acute phase symptoms of typhoid fever can be reproduced in mice by systemic administration of the typhoid toxin, but not with a catalytically-dead mutant toxin^3^. This highlights the importance of understanding the mode of action of CdtB on host cells.

CdtB shares structural and functional homology with DNase I and displays nuclease activity, observed *in vitro* by plasmid digestion or in mammalian cells by chromatin fragmentation^2^,^7^,^8^. As CdtB induces DNA double-strand breaks (DSBs), intoxication of human cells with CDT is accompanied by DSB signaling through the ATM-dependent phosphorylation of H2AX (referred to as γH2AX) and the recruitment of DSB-processing factors to damaged sites, including the MRN complex components and 53BP1^9^,^10^,^11^,^12^. The CDT-dependent activation of the ATM pathway promotes cell cycle arrest and eventually apoptotic cell death when the cell encounters excessive damage^13^,^14^.

However, several evidence challenges the model of direct DSB induction by CdtB. First, plasmid digestion by CdtB predominantly results in single-strand breaks (SSBs)^9^,^15^. Furthermore, we have shown that decreasing the CDT concentration to moderate doses (less than 1 ng/ml) induces primary DNA lesions, presumably SSBs, before DSB formation during S-phase^12^. These replication-dependent DSBs accumulate over time in proliferating cells, in contrast to the massive and rapid DSB induced by high doses of CDT (over 1 μg/ml) on both proliferating and non-proliferating cells^9^,^12^. Thus, we hypothesized that these two dose-dependent modes of CDT-induced DSB formation may activate different cellular pathways.

As mammalian cells experience thousands of DNA lesions each day, they have evolved DNA repair mechanisms to maintain genomic integrity^16^. While being partly interconnected, each repair pathway responds to specific types of DNA lesions (Table 1). Altered bases are processed by base excision repair (BER) while bulky adducts are repaired through the nucleotide excision repair (NER). SSBs, arising directly by disintegration of the oxidized sugar or indirectly as intermediates of BER, are repaired by SSB repair (SSBR)^17^. DSB management involves two major mechanisms^18^: Non-homologous end joining (NHEJ), active throughout the cell cycle, directly ligates two double-stranded DNA ends without any sequence homology requirement, whereas Homologous recombination (HR) restores DNA integrity through homology search on an undamaged template. As sister chromatid is generally used as the homologous template, HR is restricted to S and G2 cells, and, contrary to NHEJ, allows the restart of collapsed replication forks^19^. Finally, interstrand crosslink (ICL) is processed by the Fanconi Anemia (FA) pathway, which is also involved in replication fork stability^20^.

To understand which repair pathways are involved in response to CDT, a genetic screen was performed in budding yeast and identified HR as the only mechanism able to repair the CdtB-induced DNA lesions^21^. In human cells, the HR role was confirmed^12^,^22^, particularly in replication-dependent DSBs, while resistance to CDT-induced direct DSBs involved another repair pathway, presumably NHEJ^12^.

Here, we adapted the Multicolor Competition Assay (MCA)^23^, based on human cells depleted in each of the main DNA repair pathways (Table 1), to study the repair mechanisms involved in the cellular response to genotoxic treatments. The implication of each DNA repair mechanism involved in CDT resistance was confirmed with classical genetic models of DNA repair pathways. Our data substantiate the importance of the DSB repair pathways for cells to survive CDT intoxication, and demonstrate for the first time the role of SSBR. Moreover, we analyzed the functional relationship between HR, NHEJ and SSBR to respond to CDT genotoxicity. Finally, we underline the importance of the replicative stress response and identify the FA pathway as being essential following a CDT treatment. Altogether, these findings depict a global view of the DNA repair pathways involved in the resistance to CDT-mediated genotoxicity, enabling a deeper understanding of the CdtB mode of action.

## Results

### HR processing of CDT-mediated DSBs is confirmed by the Multicolor Competition Assay

**Figure 1:**
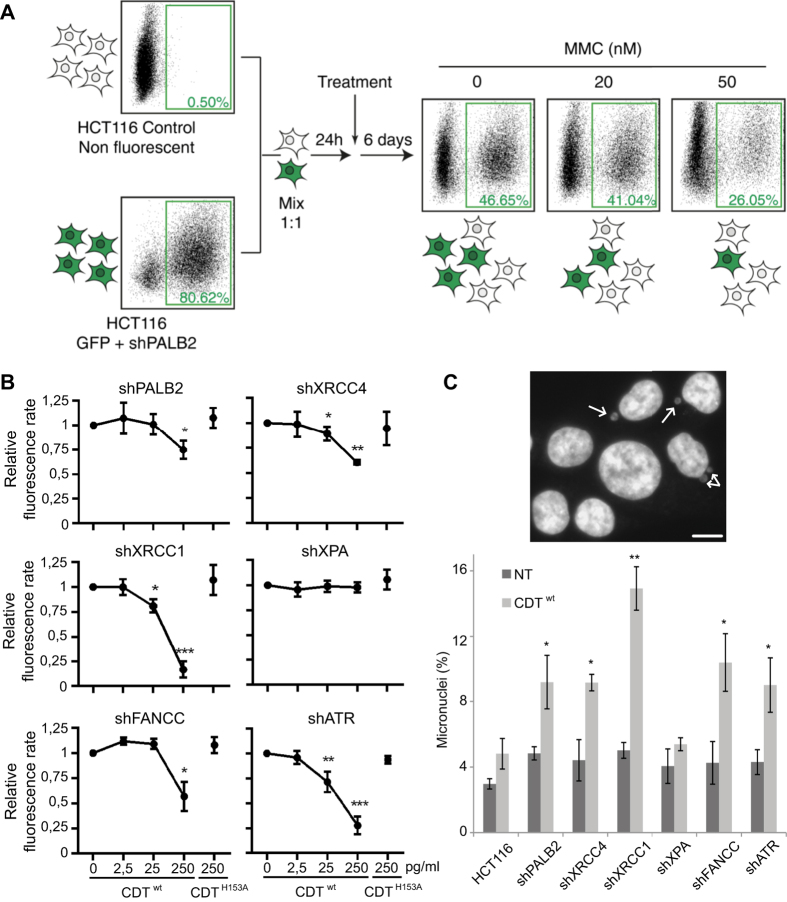
Principle and results of the MCA assay after CDT exposure. (**A**) Schematic representation of the Multicolor Competition Assay (MCA) exemplified by the validation of the shPALB2 cell line. The shRNA-mediated down-regulation of PALB2 is coupled to the green fluorescent protein (GFP) expression. Cells were co-cultured with non-fluorescent control cells with a ratio 1:1 and, after 1 day, treated with 0, 20 or 50 nM of Mitomycin C (MMC) for 6 days. MMC induces a dose-dependent decrease of the fluorescence rate, indicating a sensitization of the shPALB2 cell line. (**B**) MCA analysis in HCT116 cells expressing a shRNA directed against PALB2, XRCC4, XRCC1, XPA, FANCC or ATR. Cells were treated for 6 days with increasing doses of CDT^wt^ or with 250 pg/ml of CDT^H153A^. Data are expressed as the mean ± SD of at least 3 independent experiments. Statistics were calculated by paired Student’s t-test (*P < 0.05; **P < 0.01; ***P < 0.001). (**C**) Micronucleus frequency in HCT116 cells presented in (**B**) after chronic exposure to CDT^wt^. Upper panel shows a representative image of HCT116 cells chronically exposed to 25 pg/ml of CDT^wt^ for 2 to 3 weeks. Scale bar = 20 μm. The frequency of cells with micronuclei (white arrows) was quantified by fluorescence visualization after DAPI staining (lower panel). Data are expressed as the mean ± SD of 3 experiments. Statistics were calculated by paired Student’s t-test (*P < 0.05; **P < 0.01).

To decipher which DNA repair mechanisms are required to survive CDT intoxication, we established fluorescent human stable cell lines deficient for each repair pathway from the HCT116 p53^−/−^ background^24^. The p53 deficiency confers a slight resistance to CDT but prevents the DNA-damage induced G1 block (Fig. S1), enabling the S-phase dependent repair processing. Then we developed a test based on the Multicolor Competition Assay (MCA)^23^. Basically, fluorescent DNA repair defective cells are co-cultured with the non-fluorescent parental control cells and subjected to CDT intoxication (Fig. 1A). The ratio of fluorescent cells is compared to the untreated condition, used as a control for the relative cell growth. If CDT exposure induces DNA damage, one or more cell line will present a proliferation defect.

As HR deficient cells are hypersensitive to CDT^12^,^21^,^22^,^25^, we decided to validate the MCA strategy by confirming the role of HR. The shRNA depletion of PALB2 (Fig. S2A), an essential HR factor^26^, led to a dose-dependent reduction of the fluorescent ratio with MMC (Fig. 1A), a chemical agent known to induce ICL and HR processing^27^. We next performed MCA on shPALB2 cells treated with an active toxin (CDT^wt^) or a catalytically-dead mutant (CDT^H153A^) as a control^7^. The fluorescence ratio decreases in CDT^wt^ treated cells, demonstrating that the impairment of HR, through PALB2 down-regulation, leads to CDT sensitization (Fig. 1B). CDT^H153A^ exposure did not induce any proliferation defect. Thus, our results show that PALB2 is important to respond to CDT, confirming that HR can repair CDT-mediated DSBs and validating the MCA approach.

MCA was then conducted on isogenic cell lines deficient for NHEJ (shXRCC4), SSBR (shXRCC1), NER (shXPA), FA pathway (shFANCC) or replicative stress signaling (shATR). The shRNA-mediated gene knockdown has been confirmed for all cell lines (Fig. S2). Except for shXPA cells, all deficient cell lines exhibit a weaker growth rate compared to their parental counterpart, after CDT^wt^ but not after CDT^H153A^ (Fig. 1B). Moreover, chronic exposure to sublethal dose of CDT^wt^ induces micronucleus formation in all DNA repair defective cells apart from shXPA, indicative of enhanced genetic instability (Fig. 1C). These results indicate that cell survival after CDT treatment necessitates multiple repair mechanisms, but not NER, and involves replicative stress signaling.

### NHEJ involvement in response to CDT-induced DSBs

**Figure 2:**
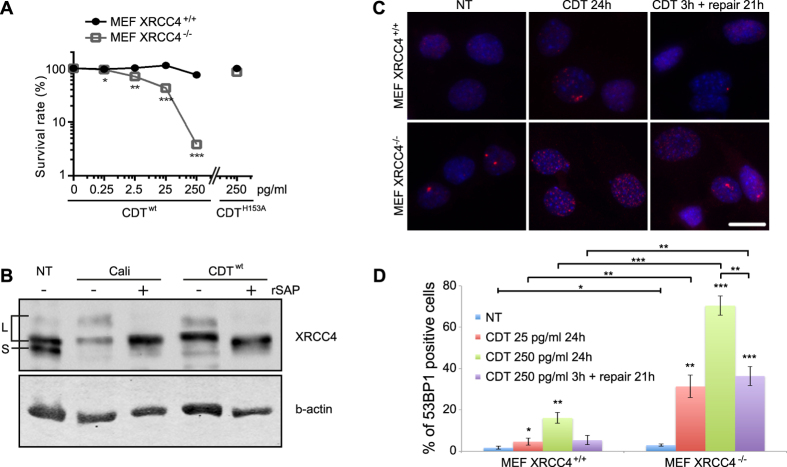
NHEJ is necessary to survive CDT-induced DSBs. (**A**) Clonogenic survival of XRCC4^+/+^ and XRCC4^−/−^ MEFs exposed to CDT^wt^ or CDT^H153A^. Results present the mean ± SD of at least 3 independent experiments. Statistics were calculated by unpaired Student’s t-test (*P < 0.05; **P < 0.01; ***P < 0.001). Graph in linear scale is presented in Fig. S9. (**B**) XRCC4 immunoblots of soluble extracts from HeLa cells treated for 1 hour with 5 nM of Calicheamicin-γ1 (Cali), or for 8 hours with 250 ng/ml of CDT^wt^. Extracts were treated or not with Shrimp Alkaline Phosphatase (rSAP) for 30 minutes at 37 °C. NT: non-treated cells. L indicates the long (phosphorylated) forms and S the short form of XRCC4. Lamin A is shown as a loading control. Full-length blots are presented in Fig. S8. (**C**) Representative images of 53BP1 immunostaining in XRCC4^+/+^ and XRCC4^−/−^ non-treated (NT) MEFs, treated with 25 pg/ml of CDT^wt^ for 24 h or for 3 h followed by a 21 h recovery time (repair 21 h). Scale bar = 20 μm. (**D**) Quantification of XRCC4^+/+^ and XRCC4^−/−^ MEFs positive for 53BP1 foci formation, after CDT^wt^ exposure for the indicated doses and times. NT: non-treated cells. Data are expressed as the mean ± SD of at least 3 independent experiments. Statistics were calculated by unpaired Student’s t-test (*P < 0.05; **P < 0.01; ***P < 0.001).

NHEJ constitutes the primary DSB repair pathway in mammalian cells^28^, with XRCC4 implicated in the ligation core complex^29^. To strengthen the MCA data obtained on shXRCC4 cells, XRCC4^−/−^ MEFs were tested for CDT sensitivity (Fig. 2A). XRCC4^−/−^ MEFs are around 20-fold more sensitive than control cells when exposed to 250 pg/ml of CDT^wt^. These effects depend on the CdtB nuclease activity, as MEF cells intoxicated with CDT^H153A^ are as viable as untreated cells.

DSB formation induces XRCC4 phosphorylation, illustrating an activation of NHEJ^30^. Compared to untreated or CDT^H153A^ treated cells, HeLa cells exposed to the DSB-inducing agent calicheamicin-γ1 or to CDT^wt^ accumulate slower mobility forms of XRCC4 (L forms) rather than the short form (S form) (Fig. 2B). These L forms represent phosphorylated XRCC4, as they are sensitive to phosphatase or Wortmaninn (Fig. S3), a general inhibitor of phosphoinositide 3-kinases, among which DNA-PK and to a lesser extend ATM have been shown to target XRCC4^31^. These results demonstrate the signaling of the CDT-induced DSB through XRCC4 phosphorylation, a characteristic of NHEJ processing.

To investigate the importance of NHEJ for managing the CDT-induced DNA lesions, XRCC4^+/+^ and XRCC4^−/−^ MEFs were exposed to 250 pg/ml of CDT for 24 h, or subjected to a 3 h treatment followed by a 21 h recovery time. DSB accumulation was monitored through 53BP1 recruitment to damaged loci (Fig. 2C). Without treatment, only a minor part of MEFs exhibits 53BP1 foci for both cell types, indicating that these cells do not suffer detectable level of endogenous DSBs (Fig. 2C,D). After 24 h of CDT, only 16% of XRCC4^+/+^ MEFs accumulate 53BP1 foci, compared to 70% of XRCC4^−/−^ MEFs (Fig. 2D), demonstrating that NHEJ impairment drastically enhances CDT-induced DSB formation. Altogether, these data show that NHEJ is implicated in the repair of CDT-induced DSBs.

### SSBR is important to respond to CDT-related genotoxicity

**Figure 3:**
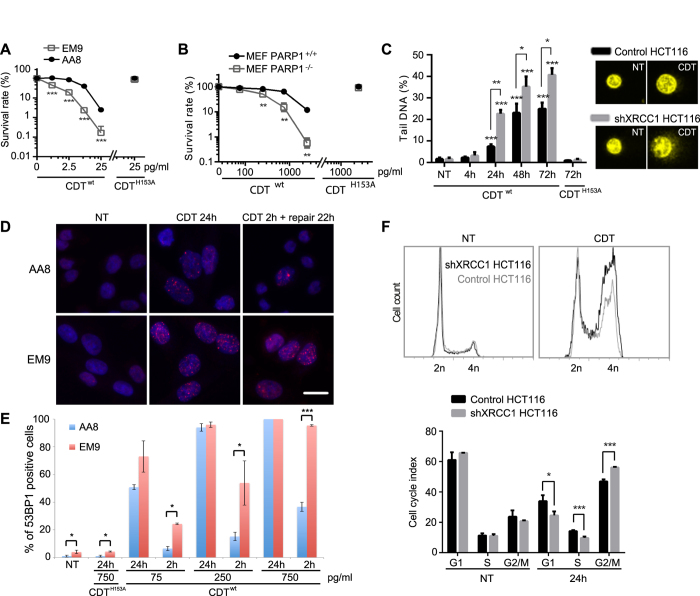
SSBR is essential in the response and repair of CDT-induced DNA damage. (**A,B**) Clonogenic survival of EM9 (XRCC1^−/−^) and AA8 (XRCC1^+/+^) CHO cells (**A**) or PARP1^+/+^ (wt) and PARP1^−/−^ MEFs (**B**) after a treatment with CDT^wt^ or CDT^H153A^. Results represent the mean ± SD of at least 3 independent experiments. Statistics were calculated by unpaired Student’s t-test (**P < 0.01; ***P < 0.001). Graphs in linear scales are presented in Fig. S9. (**C**) Alkaline COMET assays on HCT116 control and shXRCC1 cells non-treated (NT) or treated for the indicated times with 25 ng/ml of CDT^wt^ or CDT^H153A^. Left panel: Tail DNA percentage. Data are the mean ± SD of at least 3 independent experiments. Statistics were calculated by unpaired Student’s t-test (*P < 0.05; **P < 0.01; ***P < 0.001). Right panel: representative images of non-treated cells (NT) or cells treated with 25 ng/ml of CDT^wt^ for 24 h and evaluated with alkaline COMET assays. (**D**) Representative images of 53BP1 immunostaining in AA8 (XRCC1^+/+^) and EM9 (XRCC1^−/−^) non-treated (NT) CHO cells, treated with 75 pg/ml of CDT^wt^ for 24 h or for 2 h followed by a 22 h recovery time (repair 22 h). Scale bar = 20 μm. (**E**) Quantification of AA8 (XRCC1^+/+^) and EM9 (XRCC1^−/−^) CHO cells positive for 53BP1 foci formation after CDT^wt^ or CDT^H153A^ exposure at the indicated doses for 24 h or for 2 h followed by a 22 h recovery time (2 h). NT: non-treated cells. Data are expressed as the mean ± SD of at least 3 independent experiments. Statistics were calculated by unpaired Student’s t-test (*P < 0.05; ***P < 0.001). (**F**) Cell cycle analysis by flow-cytometry of HCT116 control and shXRCC1 cells non-treated (NT) or exposed for 24 h to CDT^wt^ (0.75 ng/ml). Upper panel: Graphs show the cell cycle profiles obtained for one representative experiment. Lower panel: Cell cycle index. Data represent the mean ± SD of at least three independent experiments. Statistics were calculated by unpaired Student’s t-test (*P < 0.05; ***P < 0.001).

We then aimed to clarify the consequences of a SSBR defect after CDT treatment. MCA pointed out the crucial role of XRCC1, an essential SSBR protein^32^, during repair of CDT-mediated DNA lesions (Fig. 1B). To strengthen this observation, CDT sensitivity was monitored in the XRCC1^−/−^ CHO cell line (EM9) compared to the corresponding control (AA8) (Fig. 3A), or in MEFs knocked-out for PARP1, another SSBR key protein (Fig. 3B)^33^. Cells deficient for XRCC1 or PARP1 displayed a higher sensitivity than their wild-type counterpart, for all tested CDT^wt^ concentrations, but not for CDT^H153A^. Therefore, SSBR clearly contributes to survival following CDT-induced DNA damage, favoring the assumption that SSBs are the primary lesions induced by CDT.

Then, an alkaline Comet assay was performed on shXRCC1 HCT116 cells to reveal the overall DNA damage rate, including SSBs and DSBs. No significant change was observed between the control and the XRCC1-depleted cells exposed to CDT^H153A^ or left untreated (Fig. 3C), showing that neither XRCC1 knockdown nor CDT^H153A^ induces detectable DNA lesions. Treatment with 25 ng/ml of CDT^wt^ provokes a time-dependent increase of tail-DNA level in both cell populations, which seems to reach a plateau after 48 h. This suggests that the toxin remains active for at least two days, as stated earlier^22^ and confirmed here (Fig. S4). However, shXRCC1 cells exhibit more tail-DNA level, as soon as 24 h of CDT^wt^ treatment (22.6%), compared to control cells (7.4%). Thus, cells lacking XRCC1 accumulate more damage, which may illustrate a lower repair capacity of CDT-lesions in SSBR deficient cells. Next, XRCC1^−/−^ EM9 and AA8 control cells were subjected to different doses of CDT, for 24 h or for 2 h followed by 22 h of recovery time, and DSB accumulating cells have been quantified through 53BP1 immunofluorescence (Fig. 3D,E). In absence of treatment, EM9 cells exhibit a slight but statistically significant increase in 53BP1-positive cells compared to AA8, demonstrating that impairment of XRCC1-dependent repair of endogenous DNA lesions can result in DSB. As shown earlier (Fig. 3C), CDT^H153A^ exposure does not lead to DSB formation. After 24 h of CDT^wt^, the ratio of 53BP1-positive cells is not statistically different between AA8 and EM9 in our experimental settings (Fig. 3E). However, when cells are allowed to repair the CDT-induced DNA lesions for 22 h, EM9 cells exhibit more 53BP1-positive cells for all tested doses of CDT^wt^, suggesting that the repair defects caused by XRCC1-knockout result in DSB persistence. Altogether, these data illustrate the consequence of SSBR deficiency in the accumulation of DSB after CDT.

As CDT-exposed XRCC1-deficient cells suffer more unrepaired DSBs, the DNA damage-dependent cell cycle arrest should be more important compared to control cells. Indeed, after 24 h of CDT^wt^ intoxication, the accumulation of G2/M cells is significantly more pronounced for shXRCC1 HCT116 cells, suggesting that the magnitude of G2 arrest in response to CDT reflects the level of unrepaired DNA lesions (Fig. 3F). Taken together, these data demonstrate that defective SSBR aggravates the cellular outcome of CDT exposure, with an accumulation of unrepaired DSBs associated with enhanced cell cycle arrest. In conclusion, our findings establish for the first time the crucial role of SSBR for cells to survive CDT-induced genotoxicity.

### Interplay between SSBR, NHEJ and HR in response to CDT

**Figure 4:**
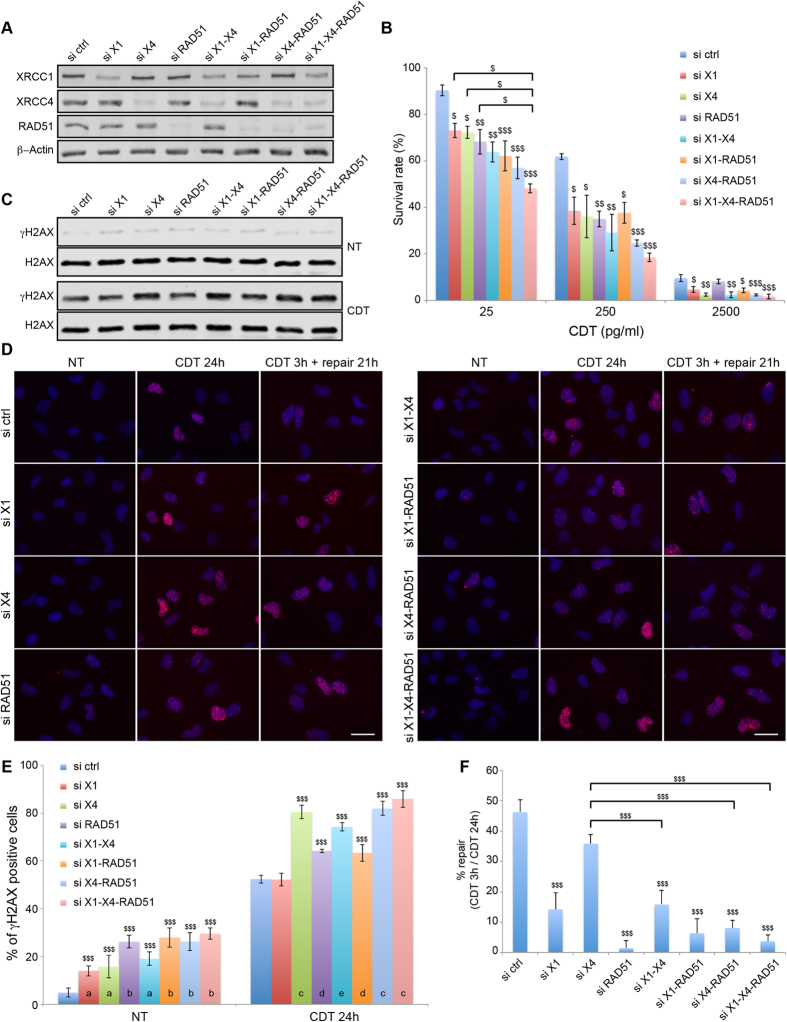
Epistatic studies of XRCC1, XRCC4 and RAD51 in response to CDT. (**A**) HeLa cells were transfected with scramble (ctrl), XRCC1 (X1), XRCC4 (X4) and/or RAD51 siRNA and soluble cell extracts were prepared to assess the level of indicated proteins with specific antibodies by Western blot analyses. β-Actin is shown as a loading control. Full-length blots are presented in Fig. S8. (**B**) Clonogenic survival of HeLa cells transfected with scramble (ctrl), XRCC1 (X1), XRCC4 (X4) and/or RAD51 siRNA and exposed to CDT^wt^. Data represent the mean ± SD of at least three independent experiments. Statistics were calculated by one-way ANOVA (^$^P < 0.05; ^$$^P < 0.01; ^$$$^P < 0.001). (**C**) Western bolt analyses of soluble extracts from Hela cells transfected with scramble (ctrl), XRCC1 (X1), XRCC4 (X4) and/or RAD51 siRNA and treated or not with 250 pg/ml of CDT^wt^ for 24 h. Full-length blots are presented in Fig. S8. (**D**) Representative images of γH2AX immunostaining in HeLa cells transfected with scramble (ctrl), XRCC1 (X1), XRCC4 (X4) and/or RAD51 siRNA, and left non-treated (NT) or treated with 250 pg/ml of CDT^wt^ for 24 h or for 3 h followed by a 21 h recovery time (repair 21 h). Scale bar = 20 μm. (**E**) Quantification of HeLa cells from (**C**) positive for γH2AX foci formation, non-treated (NT) or treated with 250 pg/ml of CDT^wt^ for 24 h. Data are expressed as the mean ± SD of at least 3 independent experiments. Statistics were calculated by one-way ANOVA (^$$$^P < 0.001). Conditions with the same letter at the bottom of the bars are not statistically different. (**F**) Quantification of the repair efficiency in HeLa cells from (**C**), calculated as the percentage of the decrease in the proportion of γH2AX positive cells from the condition CDT 3 h + repair 21 h compared to the condition CDT 24 h. Data are expressed as the mean ± SD of at least 3 independent experiments. Statistics were calculated by one-way ANOVA (^$$$^P < 0.001). Comparison among data were done using Tukey’s HSD *Post-hoc* test.

To evaluate the relative importance of SSBR, NHEJ and HR to survive CDT-mediated genotoxicity, HeLa cells were impaired for one, two or three pathways through siRNA-mediated depletion of XRCC1, XRCC4 and/or RAD51 (Fig. 4A). First, a clonogenic assay has been performed to assess CDT-sensitivity (Fig. 4B). At 25 or 250 pg/ml, control cells are statistically less sensitive to CDT than cells depleted for one or more repair pathways, corroborating the MCA results. Interestingly, down-regulation of XRCC1, XRCC4 or RAD51 alone induces a less pronounced survival defect after 25 pg/ml of CDT than when all three proteins are depleted, pointing out the cumulative effect of SSBR, NHEJ and HR impairment on CDT-mediated genotoxicity. At 2500 pg/ml, RAD51 deficiency does no longer sensitize HeLa cells to CDT, which is in agreement with our previous report showing that RAD51 is not recruited to DSBs induced at 2500 pg/ml of CDT^12^, suggesting that HR is not essential to repair direct DSBs.

Then, we investigated the consequences of XRCC1, XRCC4 and/or RAD51 knockdown on DSB induction through γH2AX accumulation after a 250 pg/ml treatment of CDT for 24 h (Fig. 4C). CDT exposure induces γH2AX accumulation that is greater when XRCC4 is down-regulated, alone or in combination. Therefore, the global DSB level induced by CDT seems more particularly regulated by NHEJ. Next, γH2AX induction has been monitored by immunofluorescence after exposure to 250 pg/ml of CDT for 24 h or for 3 h followed by 21 h of recovery (Fig. 4D). After 24 h of CDT, control and XRCC1 deficient cells display around 50% of γH2AX positive cells (Fig. 4E). The proportion of damaged cells increases up to 80 and 65% after depletion of XRCC4 and RAD51, respectively. XRCC1 loss does not enhance the bulk of DSB-accumulating cells in XRCC4 and RAD51 deficient cells. Furthermore, RAD51 or XRCC1 knockdown does not aggravate the phenotype of XRCC4 deficient cells. Thus, depletion of XRCC4, and to a lesser extend depletion of RAD51, induces an augmentation of DSB-accumulating cells after a 24 h CDT exposure, and these two responses are not cumulative. Alternatively, XRCC1 knockdown does not induce more γH2AX signal. In order to estimate the defects caused by XRCC1, XRCC4 and/or RAD51 deficiencies in the repair of the CDT-mediated DSBs, the decrease of the γH2AX positive-cells population has been calculated between the long exposure (24 h) and the short exposure to CDT (3 h) followed by a 21 h recovery time (Fig. 4F). In control cells, 46% of the γH2AX positive cells from the long exposure condition do no longer accumulate γH2AX signal 21 h after the short exposure. We infer that 46% of the damaged cells have repaired the CDT-mediated DSBs during the recovery time. Interestingly, only 14% of the XRCC1 deficient cells have lost the γH2AX staining between the long and short exposure to the toxin, implying that this lower repair capacity could explain the CDT sensitivity (Fig. 4B). The repair rate in the absence of XRCC4 is not statistically different from the control cells, suggesting that the DSB accumulation caused by NHEJ impairment can be repaired through other pathways (Fig. 4F). Indeed, depleting XRCC1 and/or RAD51 significantly decreases the repair capacity of the XRCC4 deficient cells. On the other hand, RAD51 depletion results in a drastic loss of the CDT-induced DSB repair capacity, as the proportion of γH2AX positive cells is similar between the long and short CDT-exposures. This strongly supports that DSBs directed to HR processing cannot be repaired by a compensatory mechanism. To summarize, the XRCC1-, XRCC4- and RAD51-dependent pathways play different roles in the management of the CDT-mediated DSBs. The global DSB accumulation induced by CDT is mainly hampered by NHEJ, but these lesions can be repaired through XRCC1- or RAD51-dependent mechanisms. Alternatively, other CDT-mediated DSBs are strictly processed by HR.

### The Fanconi anemia pathway is activated in response to CDT

**Figure 5:**
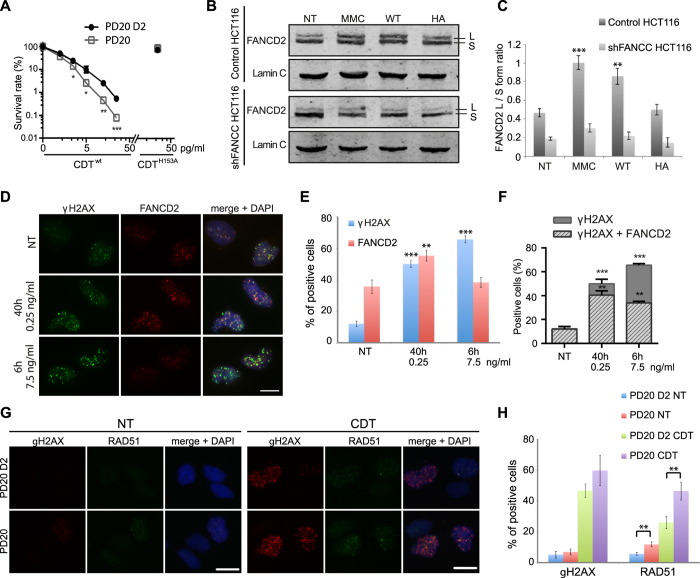
The Fanconi Anemia pathway is implicated in response to CDT-mediated DNA damage. (**A**) Clonogenic survival of PD20 and PD20 D2 cells after a CDT^wt^ or CDT^H153A^ treatment. Results are the mean ± SD of at least 3 independent experiments. Statistics were calculated by unpaired Student’s t-test (*P < 0.05; **P < 0.01; ***P < 0.001). Graph in linear scale is presented in Fig. S9. (**B**) FANCD2 immunoblots of soluble extracts from control or shFANCC cells non-treated (NT), treated for 6 h with 3 μM of MMC, or with 25 ng/ml of CDT^wt^ (WT) or CDT^H153A^ (HA). L indicates the long (monoubiquitylated) form and S the short form of the FANCD2 proteins. Lamin C is shown as a loading control. Full-length blots are presented in Fig. S8. (**C**) Quantification of the FANCD2 L form/S form ratio from (**B**). Results represent the mean ± SD of at least 3 independent experiments. Statistics were calculated by unpaired Student’s t-test (**P < 0.01; ***P < 0.001). (**D**) Representative images of FANCD2 and γH2AX immunostaining in HeLa non-treated (NT) cells, treated for 40 h with 0.25 ng/ml or for 6 h with 7.5 ng/ml of CDT^wt^. Scale bar = 20 μm. (**E**) Quantification of HeLa cells positive for FANCD2 or γH2AX from (**D**). Results represent the mean ± SD of at least three independent experiments. Statistics were calculated by unpaired Student’s t-test (**P < 0.01; ***P < 0.001). (**F**) Quantification of FANCD2 positive or negative cells in the γH2AX positive population from (**D**). Results represent the mean ± SD of at least three independent experiments. Statistics were calculated by unpaired Student’s t-test (**P < 0.01; ***P < 0.001). (**G**) Representative images of γH2AX and RAD51 immunostaining in PD20 and PD20 D2 cells non-treated (NT) or treated for 24 h with 125 pg/ml of CDT^wt^. Scale bars = 20 μm. (**H**) Quantification of PD20 or PD20 D2 cells positive for γH2AX or RAD51 foci formation from (**D**). Results represent the mean ± SD of at least three independent experiments. Statistics were calculated by unpaired Student’s t-test (**P < 0.01).

The requirement of the FA pathway to survive CDT has first been validated by MCA (Fig. 1B) on cells depleted for FANCC, component of the FA core complex^34^. Similarly, FANCD2-deficient fibroblasts (PD20) displayed reduced clonogenic survival relative to their FANCD2-complemented counterpart (PD20-D2) after CDT^wt^, and not CDT^H153A^ (Fig. 5A). Moreover, FANCD2 knockdown sensitizes HeLa cells to CDT (Fig. S5). This indicates the involvement of the FA pathway to overcome the replicative stress induced by the CDT-mediated DNA damage.

The core complex-dependent monoubiquitylation of FANCD2 is a marker of FA pathway activation^20^. When HeLa cells are intoxicated with CDT^wt^ or with MMC, FANCD2 monoubiquitylation is observed by Western blot (Fig. S6), through the increase of the FANCD2 L/S form ratio (Fig. 5B,C). In contrast, CDT^H153A^ does not impact the FANCD2 L/S ratio. FANCD2 L form accumulation is severely impaired in cells lacking FANCC, showing that the CDT^wt^-induced FANCD2 monoubiquitylation requires an intact FA pathway. Thus, CDT-induced DNA lesions promote FA pathway activation through FANCD2 monoubiquitylation.

Once monoubiquitylated, FANCD2 is recruited to chromatin damaged loci^35^. To investigate FANCD2 mobilization to the CDT-induced DSBs, that can be either direct (high doses) or replication-dependent (moderate doses), HeLa cells were subjected to two treatments (Fig. 5D). When exposed to 7.5 ng/ml of CDT^wt^ for 6 h to induce direct DSBs, 65% of cells accumulate γH2AX foci (Fig. 5E). However, the proportion of FANCD2-positive cells did not increase compared to untreated cells. In contrast, a 40 h treatment with 0.25 ng/ml of CDT^wt^ results in 50% of γH2AX positive cells, these DSBs being mostly cell cycle-dependent and thus generated during replication, as we previously reported^12^. Indeed, 55% of cells also accumulate FANCD2 foci, indicative of replicative stress signaling. In control cells, almost all γH2AX positive cells also accumulate FANCD2 foci, demonstrating that spontaneous DSB are mainly formed during S-phase (Fig. 5F). In the same way, 80% of cells that accumulate DSB in response to moderate dose of CDT are FANCD2 positive. In comparison, only 52% of cells undergoing CDT-induced direct DSBs exhibit FANCD2 foci. Thus, replication-dependent DSBs are managed by the FA machinery, whereas direct DSBs are not.

We previously reported that RAD51 is recruited to the replication-dependent DSBs induced by CDT^12^. To get more insight in the role of the FA pathway at these lesions, RAD51 localization was investigated in PD20 cells after a 24 h exposure to CDT 125 pg/ml (Fig. 5G). Under these conditions, PD20 and PD20-D2 control cells display comparable proportion of γH2AX-accumulating cells (Fig. 5H), representing the replication-dependent DSBs that must be processed through HR. However, the proportion of RAD51-positive cells is significantly higher in FANCD2 deficient cells (Fig. 5GH), suggesting that in absence of a functional FA pathway, there is an over-accumulation of the CDT-mediated DSBs that require RAD51 to be repaired.

### CDT related DNA damage induces the replicative stress response

**Figure 6:**
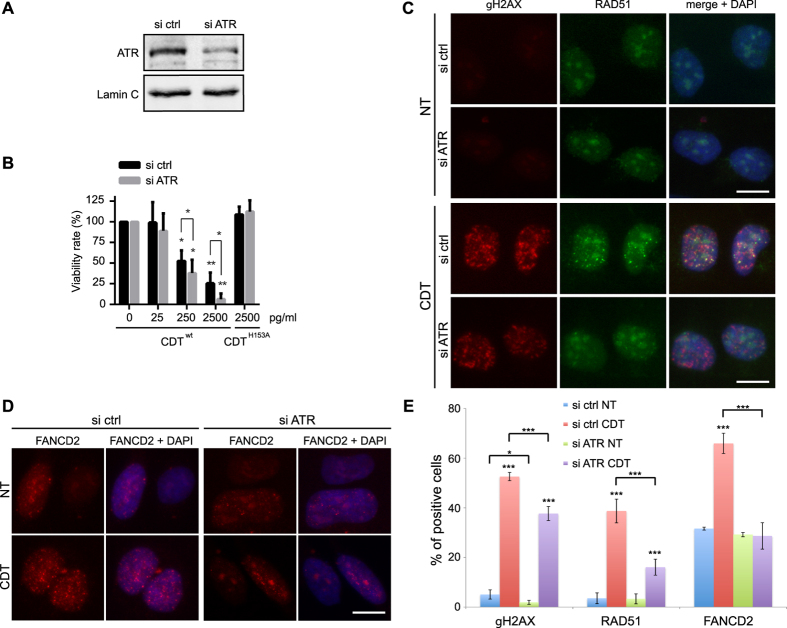
ATR requirement in the signaling and repair of the CDT-mediated DNA damage. (**A**) HeLa cells were transfected with scramble (ctrl) or ATR siRNA and soluble cell extracts were prepared to assess the ATR protein level by Western blot analyses. Lamin C is shown as a loading control. Full-length blots are presented in Fig. S8. (**B**) HeLa cells transfected with scramble (ctrl) or ATR siRNA were exposed for 5 days to CDT^wt^ or CDT^H153A^ and cell viability was analyzed by Crystal violet staining. Results represent the mean ± SD of at least three independent experiments. Statistics were calculated by unpaired Student’s t-test (*P < 0.05; **P < 0.01). (**C,D**) Representative images of γH2AX and RAD51 (**C**) or FANCD2 (**D**) immunostaining in HeLa cells transfected with scramble (ctrl) or ATR siRNA, non-treated (NT) or treated for 24 h with 250 pg/ml of CDT^wt^. Scale bars = 20 μm. (**E**) Quantification of HeLa cells transfected with scramble (ctrl) or ATR siRNA positive for γH2AX and RAD51 foci formation from (**C**) or positive for FANCD2 foci formation from (**D**). Results represent the mean ± SD of at least three independent experiments. Statistics were calculated by unpaired Student’s t-test (*P < 0.05; ***P < 0.001).

The ATR signaling pathway being crucial to face off replicative stresses^36^, we depicted the consequences of ATR depletion on the CDT-induced replication-dependent DSBs. Compared to control, ATR knockdown HeLa cells are hypersensitive to CDT^wt^, but not to CDT^H153A^ (Fig. 6A,B), confirming MCA results (Fig. 1B) and indicating that replicative stress signaling is required to respond to CDT-mediated DNA damage. Next, we asked whether ATR deficiency impedes the signaling and repair of the CDT-mediated DSBs occurring during S phase, by analyzing the formation of γH2AX, RAD51 and FANCD2 foci after a 24 h CDT-exposure (Fig. 6C,D). In HeLa cells exposed to CDT, ATR knockdown significantly reduces the accumulation of cells positive for γH2AX and RAD51, and totally abolishes the CDT-related increase of the proportion of FANCD2 positive cells (Fig. 6E). Thus, these experiments further reinforce the concept that ATR-dependent replicative stress signaling is crucial for cells to respond to the CDT genotoxic potential.

## Discussion

CDT producing bacteria have been implicated in numerous diseases, including cancer. In many cases, the CdtB catalytic activity has been shown to drive the bacterial pathogenic potential through induction of DNA damage. To better characterize the cellular processes involved in the repair of the CdtB-induced DNA lesions, we have depicted the importance of the major mammalian repair mechanisms in response to CDT. Our results compete with the classical posture supporting that resistance to CDT genotoxicity exclusively relies on the DSB repair machinery.

To deal with the different lesions that genomic DNA may suffer, cells display a battery of repair pathways^16^. The primary aim of this work was to study the importance of each of the major repair mechanisms after CDT exposure. The strategy developed here derived from MCA, successfully used to identify genes involved in DNA repair^23^,^37^. HCT116 cells were modified with shRNA to downregulate target genes (Table 1) rather than being knocked-out by endonuclease-driven gene inactivation^38^, because knockout of essential genes is lethal at the cellular level, as previously shown for ATR^39^. The decreased repair capacity of shRNA-depleted cells still induces a proliferation defect when the DNA damage level exceeds a certain threshold after genotoxic insults. Indeed, even a modest reduction of PALB2 expression level in the shPALB2 HCT116 cells was sufficient to induce a dose-dependent reduction in proliferation, after MMC or CDT, detectable by MCA. The cell viability loss of the DNA repair defective cells lines exposed to CDT has been related to enhanced genetic instability, reveled by micronucleus assay. Moreover, except for ATR that is an essential gene, all the MCA positive results were confirmed by clonogenic survival in knockout models. To conclude, the MCA strategy developed here allowed for an unambiguous overview of the repair processes required to survive CDT-induced genotoxicity, and could be used –in the future- for the screening of genotoxic compounds of unknown DNA damaging properties.

Before this study, information concerning the repair systems involved in response to CDT was limited. Two screens performed in yeast concluded that CdtB-induced DNA lesions are exclusively processed by HR^21^,^25^. Evidence of HR implication in CDT-intoxicated human cells was likewise demonstrated after RAD51 depletion and in a human pancreatic adenocarcinoma cell line deficient for BRCA2^12^,^22^. The data presented here further illustrates the role of HR by reporting the importance of PALB2 after CDT exposure, and by revealing that a subset of the CDT-related DSBs necessitates HR to be repaired. HR involvement was expected since the CDT-induced DSB formation in human cells is highly documented^9^,^10^,^11^. However, the role of NHEJ, the second canonical DSB repair system, was still controversial. If data from yeast showed that NHEJ mutants were not hypersensitive to CdtB expression^21^,^25^, one has to remind that DSB repair in yeast predominantly relies on HR processes. Our results clearly show that XRCC4, and thus NHEJ, is implicated in the resistance to CDT-induced genotoxicity by preventing DSB over-accumulation. Taken together, these data support that the two major DSB repair mechanisms, HR and NHEJ, are implicated in the resistance to the CDT-mediated DSBs.

Several evidences suggest that CdtB does not induce direct DSBs. First, comparison between yeast mutant strains hypersensitive to CdtB and direct DSB inducing agents (such as ionizing radiation or HO and EcoRI endonucleases) indicates that the CdtB-induced DSBs are atypical^25^,^40^,^41^. Different types of DNA lesions, such as SSBs or bulky lesions, hinder the progression of replication forks and cause fork collapse and DSB formation^42^. According to MCA data, inhibition of XPA, an important player of global NER^43^, does not impact the viability of CDT-treated cells, strongly supporting that CDT-mediated DNA damage does not involve bulky lesions. Therefore, CDT-induced primary DNA lesions may be SSBs, as supported by several anterior works (see ref. 44 for detail). To test this hypothesis, we studied the XRCC1-depleted cells after CDT treatment. XRCC1 is a scaffold protein that interacts with different SSBR components to stabilize and/or stimulate them^45^. As expected, human and rodent XRCC1 deficient cells were hypersensitive to CDT. Besides, yeast data showed that DNA glycosylases involved in early steps of BER are not required to survive CDT intoxication, implying that the toxin does not generate base modifications^21^,^25^. Thus, these findings strongly suggest that CdtB, through its nuclease activity, only induces DNA strand breaks.

Alkaline comet assays showed that CDT-treated cells accumulate DNA strand breaks over time, and this effect is aggravated by reduced SSBR capacity in XRCC1-deficient cells. This indicates that once internalized, CdtB continuously generates SSBs, some of which are repaired by SSBR. The unrepaired SSBs sustain the CdtB-mediated genotoxicity, underpinning the well-characterized cellular consequences of CDT intoxication that are cell cycle arrest, cell distension and cell death. Indeed, shXRCC1 HCT116 cells accumulate unrepaired SSBs, and exhibit proliferation defects, as observed by MCA, and cell distension (data not shown) compared to control cells. The CDT-mediated cell cycle arrest and apoptosis depends on ATM^13^,^14^. This implies that the CdtB-induced SSBs should be converted to DSBs to activate the ATM-dependent pathway, even if we cannot rule out the possibility that SSBs may partially mediate ATM activation^46^. XRCC1^−/−^ EM9 CHO cells accumulate more DSBs than control cells during a continuous exposure to CDT, and the subsequent DSB repair after a short treatment is attenuated by XRCC1 depletion, even in knockdown HeLa cells. This effect may result from the impairment of the XRCC1-dependent alternative DSB repair pathway^47^. Otherwise, it could reflect the defective repair of the SSBs generated after the pulse treatment by CDT, which may degenerate into DSB during S-phase, as CDT remains active in the cell for more than 48 h post-exposure and induces more DNA breaks in shXRCC1 than in control cells.

CDT can directly induce DSBs when two SSBs face each other, or indirectly during S-phase, associated with the presence of single-stranded DNA coated by RPA^12^. The later condition implicates the activation of the ATR-dependent replicative stress response^48^. CDT was shown to induce a rapid phosphorylation of ATR and CHK1 that increases over time^22^. Here, we observed a hypersensitivity to CDT in two different ATR-depleted fibroblasts cell lines. Moreover, ATR deficiency results in a diminished accumulation γH2AX, RAD51 and FANCD2 in CDT-treated cells. These results pinpoint the need for ATR-dependent replicative stress signaling to recover from the CdtB-mediated DNA damage in S-phase.

To further characterize the cellular response to the CDT-induced replicative stress, attention has been drawn to the FA pathway. Besides its well-established role in the repair of ICL, FA pathway plays a more general role in the recovery from replication stress^20^. Here we show that CDT promotes FA pathway activation through FANCD2 monoubiquitylation, strongly supporting that CDT-mediated DNA damage induce a replicative stress. This was confirmed by the hypersensitivity of FANCC and FANCD2 deficient cells to CDT, and the FANCD2 recruitment to the replication-dependent DSBs induced by CDT moderate doses. FANCD2 is highly enriched at stalled and collapsed replication forks^49^, suggesting that CDT-induced SSBs block the replication fork progression. FA proteins have been shown to protect stalled replication forks^50^, to prevent the accumulation of replication-associated DSBs^51^ and stabilize collapsed replication forks^52^. We show here an increased recruitment of RAD51 to CDT-induced lesions in absence of FANCD2, suggesting that HR processing of replication-dependent DSBs is delayed in FANCD2 deficient cells, leading to an over-accumulation of RAD51 positive cells. Alternatively, this enhanced RAD51 loading might represent its DSB repair-independent role in the stabilization and protection of stalled forks to compensate for FANCD2 depletion^50^,^53^. Thus, FA pathway seems required to overcome the SSB-mediated replication fork blockage induced by CDT.

**Figure 7:**
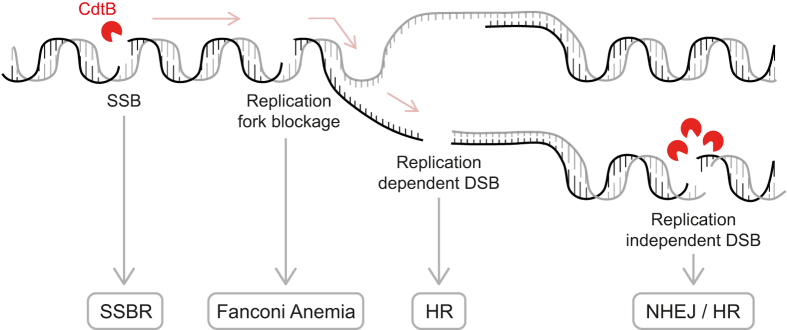
Model for repair of CDT-induced DNA damage. Moderate CDT^wt^ doses induce SSBs, repaired by SSBR. SSBs left unrepaired or produced during S-phase block the replication fork, inducing a replicative stress signaled by ATR and activating the Fanconi Anemia pathway. The stalled replication fork will eventually collapse to form a one-ended DSB, which is specifically processed by HR. On the other hand, high doses of CDT^wt^ directly induce replication-independent DSBs by creating two SSBs facing each other on the two DNA strands. These DSBs can be repaired either by HR or NHEJ.

In conclusion, we have demonstrated that cellular survival to CDT-mediated genotoxicity involves a battery of repair mechanisms. Our results emphasize the implication of the SSBR and FA pathways, favoring the model in which the unrepaired CDT-induced SSBs degenerate into replication-associated DSBs (Fig. 7, see legend for details). This model underlines the cell proliferation status to generate CDT-mediated DSBs, and demonstrates the importance of possessing functional DNA repair processes during a CDT-producing bacteria infection to counteract the mutagenic potential of this toxin. Chronic infection with *Salmonella enterica* serovar Typhi is associated with an increased risk of hepatobiliary carcinoma^54^. In mouse, chronic infection with CDT-expressing *Campylobacter jejuni* or *Helicobacter hepaticus* is associated with gastric or liver dysplasia, respectively^5^,^55^. Finally, chronic exposure of human cells to CDT promotes genomic instability with impaired DNA damage response, especially in pre-malignant backgrounds^56^,^57^. Concerning this aspect, our data suggest that the cumulative deficiencies of p53 and DNA repair pathways can result in additive but not synergistic effects. Our MCA data on p53 deficient cells have been confirmed with p53 proficient genetic models, supporting that p53 status does not notably affect the behavior of DNA repair defective cells exposed to CDT. Besides, p53 inhibition in these cells confers some resistance to CDT (Fig. S7), even with increased genetic instability, which may be a consequence of reduced apoptosis. Future studies need to further address this issue, considering more carefully the p53-dependent cell cycle arrest, because an active G1/S checkpoint could prevent S-phase entry and thus the replication-associated DSBs by CDT. Our findings provide critical insight into the molecular mechanism by which CDT acts, coupling its genotoxic effect with the proliferation status of the host cells, thereby inducing a replicative stress that could ultimately lead to genetic instability and cancer^58^. Future studies will necessarily provide further insight into the mechanisms contributing to the pathogenesis of disease involving CDT-producing bacteria, including cancer, particularly in the context of epithelia where cells are highly proliferating.

## Methods

### Cell lines and treatments

Human colon cancer cells (HCT116), HeLa cells and Mouse Embryonic Fibroblasts (MEFs) were maintained in Dulbecco’s modified Eagle’s medium (DMEM, Gibco, Life technologies). The human PD20 cell line and the FANCD2 complemented cell line (PD20 D2) were maintained in Opti-MEM (Gibco, Life technologies). Finally, AA8 and EM9 cell lines were maintained in a mixture of 50% DMEM media and 50% F-12 media. All mediums were supplemented with 10% heat-inactivated calf serum, and 0.5 mg/ml Penicillin/Streptomycin (P/S). Cells lines were grown in a humidified incubator at 37 °C with 5% CO_2_.

The Cytolethal Distending Toxins (CDT^wt^ or CDT^H153A^) were produced and purified as described previously^12^. When needed, HCT116 cells were treated with Mitomycin C (MMC) (Sigma-Aldrich), and HeLa cells were treated with 10 pM of calicheamicin-γ1 (Pfizer) or with 20 μM of Wortmannin (Sigma-Aldrich).

### Small-hairpin RNA (shRNA) knockdown

Stable cell lines defective in the different repair pathways (see Table 1 for details) were generated in HCT116 p53^−/−^ cells^24^ according to standard protocols using Mission short hairpin RNA (shRNA) lentiviral particles bearing the pLKO.1-puro vector either containing the turboGFP, TagCFP or TagRFP gene (Sigma-Aldrich). Targeted genes and shRNA sequences are listed in Table 1. Briefly, HCT116 cells were infected with lentiviral particles for 24 h, then puromycin was applied to select for lentiviral transduction. Puromycin resistant cells were diluted (limit-dilution), and fluorescent clones were selected, amplified and screened for the shRNA-mediated depletion efficiency by Western blot and RT-qPCR analysis.

### Cytometry analyses

Cell cycle analysis was done as previously reported^12^. For Multicolor Competition Assay, control cells and fluorescent shRNA cells were counted, mixed with a 1:1 ratio, and were left untreated or treated either with CDT^wt^ or CDT^H153A^. After 6 days of culture, cells were collected and the fluorescent rate was analyzed by flow cytometry with a Miltenyi MACSQuant Analyser 10 cytometer. Analysis was made with VenturyOne software (Applied Cytometry). Relative survival of shRNA-untreated cells was set to 1.

### RNA interference

Gene silencing was performed by transfection of siRNA (Sigma-Aldrich) in HeLa cells with INTERFERin^®^ according to the manufacturer’s instructions (Polyplus). Briefly, siRNA were mixed with Interferin in Opti-MEM, and incubated 10 minutes at room temperature. Then, HeLa cells were transfected with the negative control siRNA (CAUGUCAUGUGUCACAUCU-dTdT), or with siRNA directed against XRCC1 (GGAAGAUAUAGACAUUGAG-dTdT), XRCC4 (AAUCUUGGGACAGAACCUAAA-dTdT), RAD51 (CCAGAUCUGUCAUACGCUA-dTdT), FANCD2 (AACAGCCAUGGAUACACUUGA-dTdT), or ATR (CCUCCGUGAUGUUGCUUGA-dTdT). After 24 h of incubation, cells were counted and plated for further analysis.

### Crystal violet viability assay

24 h after siRNA transfection, 6 000 HeLa cells were grown for 24 h in 24-well plates before being treated with CDT for 6 h. Cells were washed and released in fresh media for 5 days. After a PBS wash, cells were fixed for 10 minutes with 10% (vol/vol) methanol/10% (vol/vol) acetic acid at room temperature. Cells were then stained for 10 minutes with 1% (vol/vol) crystal violet (Sigma-Aldrich) in methanol, washed in water and the absorbed dye was released by incubation with agitation for 1 h at room temperature in methanol containing 0.1% sodium dodecyl sulfate (SDS). The dye containing solutions were then transferred to 96-well microtiter plates, and dilutions (1:2) were prepared. The optical density (OD) at 595 nm was assessed in an Infinite 200 PRO reader (TECAN).

### Clonogenic assay

Cells were plated in triplicate at a density of 200 to 9000 cells per well in 6 wells plate. One day after seeding, cells were treated with CDT and grown for 6–12 days. For HeLa cells treated with siRNA, cells were exposed to CDT 66 h post-transfection for only 3 h before to be released in fresh media. Formed colonies were fixed and stained with a 0.25% methylene blue (Sigma) and 50% methanol solution. Colonies containing more than 50 cells were counted and the surviving rate calculated.

### Micronucleus assay

Cells with chronic and no chronic treatment were grown in 12-well plates (2 × 10^4^ cells/well) for 48 h. Cells were fixed with 4% of paraformaldehyde for 20 min, after a PBS wash. Then cells were permeabilized with 0.5% Triton X-100 in PBS for 15 min, and nuclei were stained with DAPI (100 nM).

### RT-qPCR analysis

Total RNA was extracted from cultured cells using TRIzol^®^ Reagent (Invitrogen) and RT-PCR was performed with the High-Capacity cDNA Reverse Transcription kit according to the manufacturer’s instructions (Applied Biosystems). Real-time quantitative PCR (RT-qPCR) was performed using the Power SYBR^®^ Green PCR Master Mix and an ABI Prism 7300 Sequence Detection System instrument and software (Applied Biosystems). The following gene-specific primers were used: PALB2 (forward: 5′-GGAGCTGCATAAACATTCCGTCG-3′; reverse: 5′-CTACGGAACAGGAACCTGAAGG-3′); FANCC (forward: 5′-CTGGCTCCAGACACTGAAGCAT-3′; reverse: 5′-ATTGCTCTGCCACCATCTCAGC-3′,); and TBP (forward: 5′-TGTATCCACAGTGAATCTTGGTTG-3′, reverse: 5′-GGTTCGTGGCTCTCTTATCCTC-3′) (Sigma Aldrich). TBP was chosen as an internal control. The comparative threshold cycle (2^−ΔCt^) method was used to enable the mRNA quantification of these genes. All samples were run in triplicate. After PCR, a melting curve was obtained and analyzed.

### Western blot analysis

Cells were incubated on ice for 30 min in lysis buffer (50 mM Tris-HCl pH7.5, 500 mM NaCl and 0.5% NP40) containing the Halt^TM^ Protease & Phosphatase inhibitor cocktail (Thermo Scientific) and sonicated on a VibraCell 72434 (Bioblock Scientific). Cell lysates were centrifugated and the supernatant containing total soluble proteins was kept. Chromatin and soluble fractions were then prepared as previously described^59^. Proteins were separated by SDS-PAGE and transferred to a nitrocellulose membrane (Amersham). Membranes were incubated with the primary antibody for a period of 1–16 hours. H2AX antibody was purchased from Epitomics (3522-1), γH2AX antibody from Merck/Millipore (05–636), ATR (SAB4200348), Lamin A/C (SAB4200236), β-actin (sc-47778) antibodies from Santa Cruz, XRCC1 (X0629), XRCC4 (HPA006801), XPA (SAB1406599-50UG) antibodies from SIGMA, FANCD2 (NB100-182) antibody from Novus Biologicals, and GAPDH (GTX100118) antibody from GeneTex. Secondary fluorescent antibodies (CF^TM^770 Goat anti-rabbit (20078) or anti-mouse (20077) and CF^TM^680 anti-rabbit (20067) or anti-mouse (20065), purchased from BIOTUM, were visualized on the membrane using an Odyssey Infrared Imaging Scanner (Li-Cor ScienceTec). Lamin, β-actin or GAPDH were used as internal control to normalize the protein level.

### Immunofluorescence analysis

Cells were grown on glass coverslips. After at least 24 h of culture, cells were fixed with 4% paraformaldehyde, permeabilized with 0.5% Triton X-100 and stained with primary antibodies over-night at 4 °C. 53BP1 (NB100-304) and FANCD2 (NB100-182) antibodies were purchased from Novus Biologicals, γH2AX antibody from Merck/Millipore (05–636), and RAD51 antibody from Santa Cruz biotechnology (sc-8349). Cells were washed three times with PBS Tween 0.1% and incubated with the secondary antibodies for 1 h (Rhodamine Red X (R6394) and AlexaFluor 488 Goat anti-mouse (A11017) or anti-rabbit (A11070), purchased from Invitrogen). DNA was stained with 4.6-diamino-2-phenyl indole (DAPI). Cells were counted positive for foci formation when >10 foci/nuclei were detected.

### Comet assay

Alkaline comet assay was performed as previously described^60^ with minor modification. Cells were embedded in 0.7% low melting point agarose and laid on CometAssay^®^ HT Slide (Trevigen). Fifty cells per slide and 2 slides per sample were analyzed. The extent of DNA damage was evaluated for each cell through the measurement of intensity of all tale pixels divided by the total intensity of all pixels in head and tail of comet. The median from these 100 values was calculated, and named % tail DNA.

### Statistical analysis

When not specified, results are expressed as the mean of three independent experiments. The error bars represent the standard deviation (SD). For clonogenic and crystal violet viability assays, differential effects between the two cells type for a same treatment condition were assessed by unpaired Student’s t-test. Paired and unpaired Student’s t-tests were used for MCA and immunofluorescence experiments, respectively. For comet assays, unpaired Student’s t-test was applied to compare a time point to the non-treated (NT) condition for a unique cell line, while paired Student’s t-test was used to compare values for a same time point between two cell lines. P-values < 0.05 were considered significant (indicated by asterisks): *p < 0.05; **p < 0.01, ***p < 0.001. For multiple comparisons of siRNA-treated cells, different responses of treatments were analyzed by one-way ANOVA. P-values < 0.05 were considered significant (indicated by dollars): $p < 0.05; $$p < 0.01, $$$p < 0.001. Comparison among data were done using Tukey’s HSD *Post-hoc* test.

In these experiments, when the difference between the non-treated (NT) and a treated condition was assessed, the results are indicated above the error bar of the treated condition except for clonogenic assays in which the difference between two cell lines for a same condition is indicated below the error bar. When the difference between two treated conditions was assessed, the results are indicated above a horizontal line. When multiple comparisons classify the different conditions in groups, these groups are indicated by lowercase letter at the bottom of the bars in the histograms. Statistical analyses and plots were generated using GraphPad Prism 6.0 software or Excel.

## Additional Information

**How to cite this article**: Bezine, E. *et al*. Cell resistance to the Cytolethal Distending Toxin involves an association of DNA repair mechanisms. *Sci. Rep*. **6**, 36022; doi: 10.1038/srep36022 (2016).

## Supplementary Material

Supplementary Information

## Figures and Tables

**Table 1:** Summary of the DNA repair proteins down-regulated in HCT116 cells.

Depleted protein	Pathway (DNA lesion)	Protein function	shRNA target sequence
PALB2	HR (DSB; ICL)	Forms a complex with BRCA1 and BRCA2; HR mediator	GATGCACATTGATGATTCTTA
XRCC4	NHEJ (DSB)	Interacts with Lig4 and XLF to form the DSB end ligation complex	CCTCAGGAGAATCAGCTTCAA
XPA	NER (bulky lesions)	Binds and stabilizes the open pre-incision complexes; recruits ERCC1-XPF endonuclease	CATGAGTATGGACCAGAAGAA
XRCC1	SSBR (SSB)	Scaffold protein; stabilizes and stimulates multiple enzymatic components during SSBR	CCAGTGCTCCAGGAAGATATA
ATR	Replicative stress signaling	Serine/threonine kinase involved in the replicative stress signaling	GCCAAAGTATTTCTAGCCTAT
FANCC	Fanconi Anemia (ICL)	Subunit of the FA core complex involved in FANCD2/FANCI monoubiquitylation	CACGAGATCATTGGCTTTCTT

This table depicts the down-regulated proteins, the repair pathways associated (and the processed DNA lesions), their function and the sequence targeted by the shRNA.
