# Supplementary material for: Cell resistance to the Cytolethal Distending Toxin involves an association of DNA repair mechanisms
Source: Sci Rep. 2016 Oct 24;6:36022. doi: 10.1038/srep36022 (PMC5075911; doi:10.1038/srep36022)
Supplement: Supplementary Information [file srep36022-s1.pdf]

# SUPPLEMENTARY INFORMATION

## Cell resistance to the Cytolethal Distending Toxin involves an association of DNA repair mechanisms

BEZINE E., MALAISÉ Y., LOEUILLET A., CHEVALIER M., BOUTET-ROBINET E., SALLES B., MIREY G.*, VIGNARD J.*

Toxalim, Université de Toulouse, INRA, ENVT, INP-Purpan, Université de Toulouse 3 Paul Sabatier, Toulouse, France

* These authors are co-corresponding authors.

e-mail address, telephone and fax numbers of the corresponding authors:
MIREY G.: gladys.mirey@toulouse.inra.fr; Tel: +33 582 06 63 38; Fax: +33 561 28 52 44
VIGNARD J.: julien.vignard@toulouse.inra.fr; Tel: +33 582 06 63 64; Fax: +33 561 28 52 44

## Supplementary Methods

### *PrestoBlue Cell Viability Assay*

PrestoBlue Cell Viability Assay (Invitrogen) has been performed according to the manufacturer's instructions. Briefly, HCT116 cells were grown on 96 well plates (10 000 cells/well) during 24 h. Then, cells were incubated in duplicate at three concentrations of CDT (2.5, 0.25, 0.025 µg/ml). After 72 h of treatment, cells were incubated with the PrestoBlue Cell Viability Reagent (1X) for 2 h at 37°C and absorbance (570 nm measurement wavelength, 600 nm reference wavelength) was read using an Infinite 200 PRO reader (TECAN). The percentage of cytotoxicity was determined by comparing results with non-treated cells.

### *Immunoprecipitation*

$10^7$ HCT116 cells exposed for 24 h to 250 pg/ml of $CDT^{wt}$ before to be collected and lyzed in IP250 buffer (50 mM Tris-HCl pH 7.5, 250 mM NaCl, 0.05% NP40), and soluble extract was incubated with antibodies against FANCD2 from GeneTex (GTX116037) or from Novus Biologicals (NB100-182) overnight at 4°C under constant agitation. Then, 20 µl of equilibrated Protein A Magnetic Beads (Thermo Scientific) were added for 1 h before to be washed for 5 minutes three times in IP250 buffer before to proceed to immunoblots.

## Supplementary Figure

**Fig. S1** Effects on cell viability and cell cycle arrest caused by p53 deficiency on HCT116 cells exposed to CDT.

A. Prestoblue Cell Viability Assay, clonogenic assay and MCA show that p53 deficiency increases resistance to $CDT^{wt}$ in HCT116 cells. MCA has been performed by co-culturing HCT116 $p53^{+/+}$ cells expressing GFP with non-fluorescent HCT116 $p53^{+/+}$ or HCT116 $p53^{-/-}$cells. For clonogenic assay, results present the mean ± SD of 3 experiments. Statistics were calculated by unpaired Student's t-test (*P < 0.05).

B. Cell cycle analysis by flow-cytometry of HCT116 $p53^{+/+}$ and HCT116 $p53^{-/-}$ cells non-treated (NT) or exposed for 48 h to etoposide (5 μM) or $CDT^{wt}$ (2.5 ng/ml). Graphs show the cell cycle profiles obtained for one representative experiment.

**Fig. S2** Efficiency of shRNA-mediated depletion in HCT116 stable cell lines. The mRNA expression levels in HCT116 cell lines down-regulated for PALB2 (A) or FANCC (F) were analyzed by RT-qPCR. The protein levels were analyzed by Western blot in soluble cell extracts of HCT116 cell lines down-regulated for XRCC4 (B), XPA (C), XRCC1 (D) or ATR (E).

**Fig. S3** CDT induced XRCC4 phosphorylation is inhibited by Wortmaninn.

A. XRCC4 immunoblots of soluble extracts from HeLa cells pre-exposed or not for 1 hour with 30 μM of Wortmannin (Wort) before to be treated for 1 hour with 10 pM of Calicheamicin-γ1 (Cali), or for 6 hours with 25 ng/ml of $CDT^{H153A}$ (HA) or $CDT^{wt}$ (WT). NT: non-treated cells. L indicates the long (phosphorylated) form and S the short form of XRCC4. Lamin A is shown as a loading control. Full-length blots are presented in Supplementary Figure 8.

B. Quantification of the XRCC4 L form / S form ratio from the Western blots presented in (A). Results represent the mean ± SD of at least three independent experiments. Statistics were calculated by unpaired Student's t-test (* $P < 0.05$).

**Fig. S4** Quantification of alkaline Comet assay on HCT116 cells after a 4 h pulse treatment of $CDT^{wt}$ followed by different release times. Tail DNA percentage. Data are the mean ± SD of at least 3 independent experiments (* $P < 0.05$; ** $P < 0.01$; *** $P < 0.001$).

**Fig. S5** FANCD2 depletion sensitizes HeLa cells to CDT.

A. FANCD2 protein level analyzed by Western blot in soluble cell extracts of HeLa cells treated with control or FANCD2 siRNA for 72 h. β-actin is shown as a loading control.

B. HeLa cells treated with control or FANCD2 siRNA were exposed for 5 days to $CDT^{wt}$ or $CDT^{H153A}$ and cell viability was analyzed by crystal violet staining (* $P < 0.05$; ** $P < 0.01$; *** $P < 0.001$).

**Fig. S6** Western blot with mono- and polyubiquitinylated conjugates (FK2) and FANCD2 antibodies after FANCD2 immunoprecipitation with two different FANCD2 antibodies. Immunoprecipitations on soluble extracts from HCT116 exposed to 250 pg/ml of $CDT^{wt}$ for 24 h were conducted with beads alone or two different polyclonal antibodies against FANCD2 and blotted against anti mono- and polyubiquitinylated conjugates (FK2) or FANCD2, as indicated.

**Fig. S7** Effect of p53 inhibition on cell survival of MEFs and PD20 cells exposed to CDT.

A. Clonogenic survival of $XRCC4^{+/+}$ and $XRCC4^{-/-}$ MEFs treated or not with 10 $\mu$M of Pifithrin-$\alpha$ (PFT) and exposed to $CDT^{wt}$ or $CDT^{H153A}$. Results present the mean ± SD of at least 3 independent experiments.

B. Clonogenic survival of $PARP1^{+/+}$ and $PARP1^{-/-}$ MEFs treated or not with 10 $\mu$M of Pifithrin-$\alpha$ (PFT) and exposed to $CDT^{wt}$ or $CDT^{H153A}$.

C. Clonogenic survival of PD20 D2 and PD20 cells treated or not with 10 $\mu$M of Pifithrin-$\alpha$ (PFT) and exposed to $CDT^{wt}$ or $CDT^{H153A}$. Results are the mean ± SD of at least 3 independent experiments. Statistics were calculated by unpaired Student's t-test (** $P < 0.01$; *** $P < 0.001$).

D. Cell cycle analysis by flow-cytometry of $XRCC4^{+/+}$ MEFs non-treated (NT) or treated or not with 10 $\mu$M of Pifithrin-$\alpha$ (PFT) and exposed for 24 h to $CDT^{wt}$ (2.5 ng/ml).

E. Micronucleus frequency in $XRCC4^{+/+}$, $XRCC4^{-/-}$, $PARP1^{+/+}$ and $PARP1^{-/-}$ MEFs, PD20 D2 and PD20 cells exposure to $CDT^{wt}$ for 24 to 36 h. The CDT dose was 75 pg/ml for $XRCC4^{+/+}$ and $XRCC4^{-/-}$ MEFs, 75 pg/ml for $PARP1^{+/+}$ and $PARP1^{-/-}$ MEFs, and 2.5 pg/ml for PD20 D2 and PD20 cells. Results are the mean ± SD of 3 independent experiments.

**Fig. S8** Full-length blots of the different cropped blots from this study.

**Fig. S9** Representation in linear scale of the quantification of clonogenic assays presented in Fig. 2A, 3A-B and 5A.

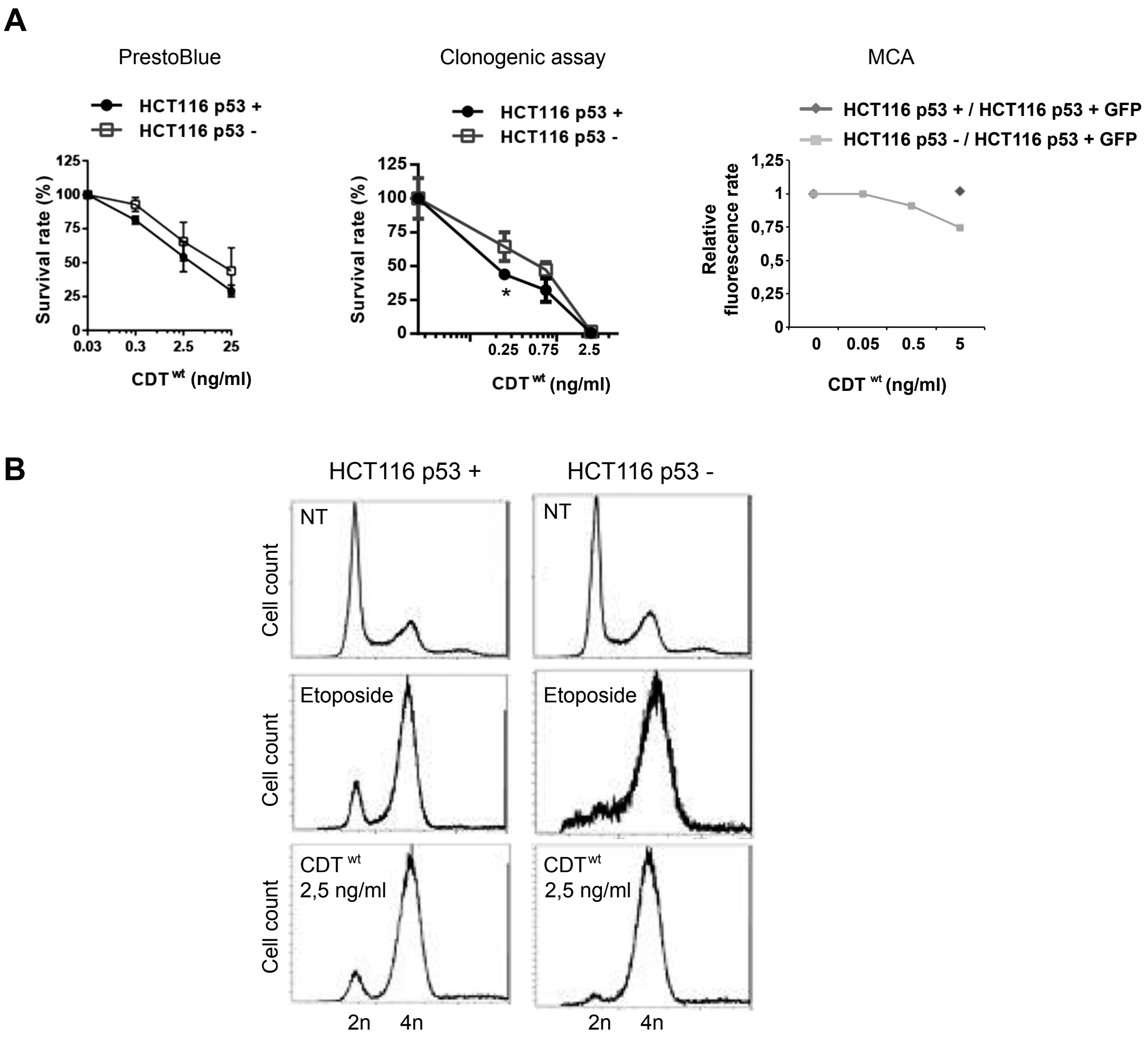


**Fig S1**. Effects on cell viability and cell cycle arrest caused by p53 deficiency on HCT116 cells exposed to CDT.

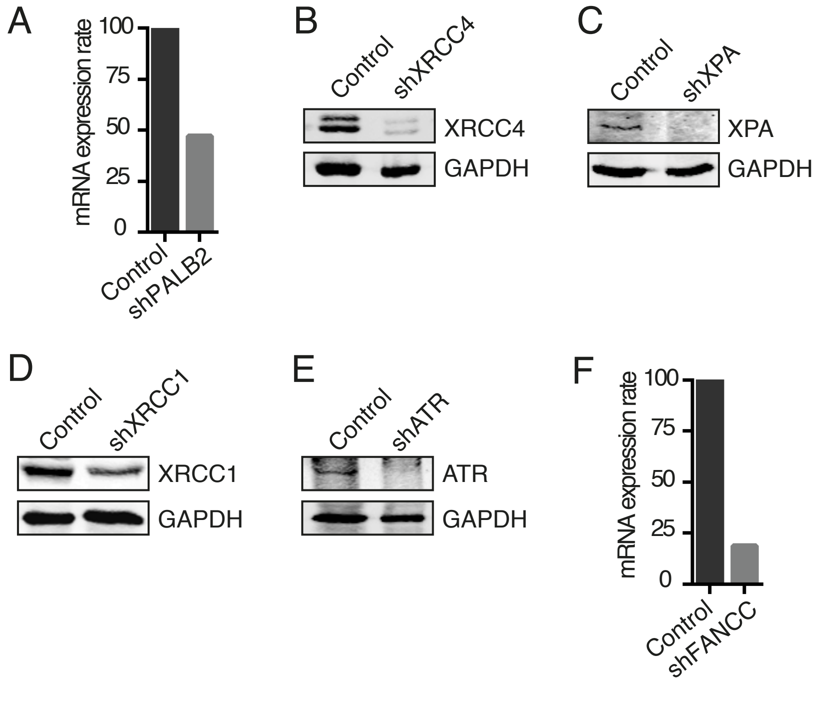


**Fig. S2** Efficiency of shRNA-mediated depletion of the proteins of interest in HCT116 stable cell lines.

**Bezine et al., 2016**

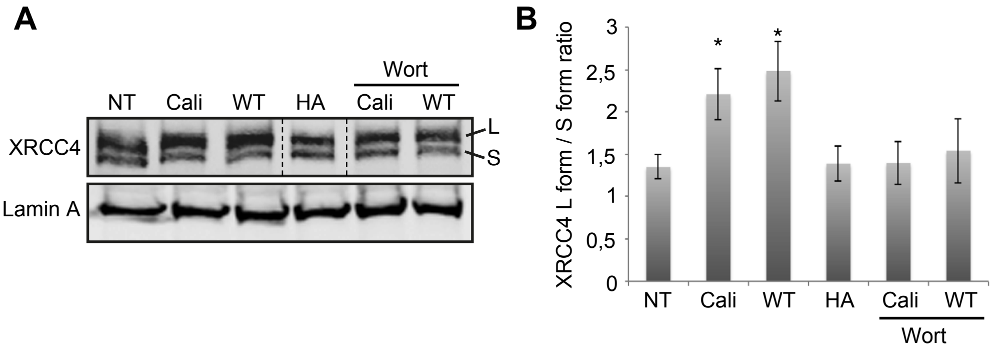


**Fig. S3** XRCC4 phosphorylation in response to CDT is inhibited by Wortmaninn

**Bezine et al., 2016**

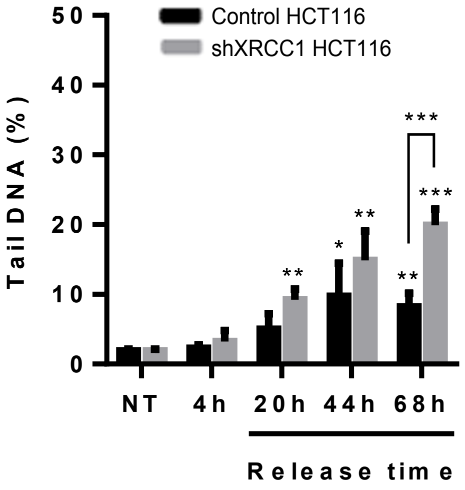


**Fig. S4** Quantification of alkaline COMET assay on HCT116 cells after a 4 h pulse treatment of CDT $^{wt}$ followed by different release times

**Bezine et al., 2016**

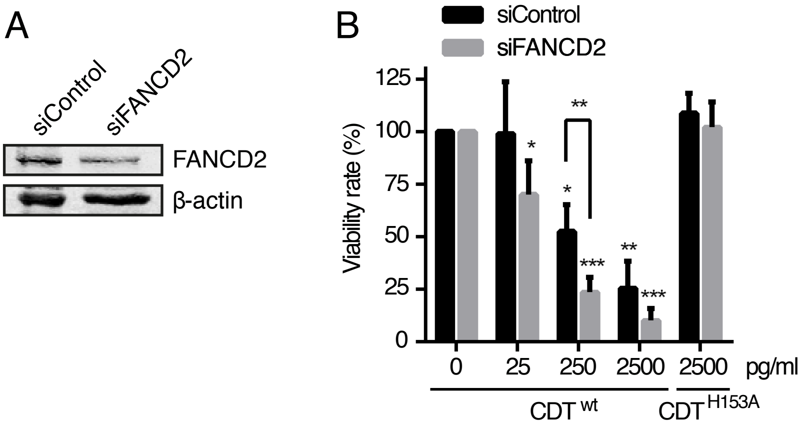


**Fig. S5** FANCD2 depletion sensitizes Hela cells to CDT

**Bezine et al., 2016**

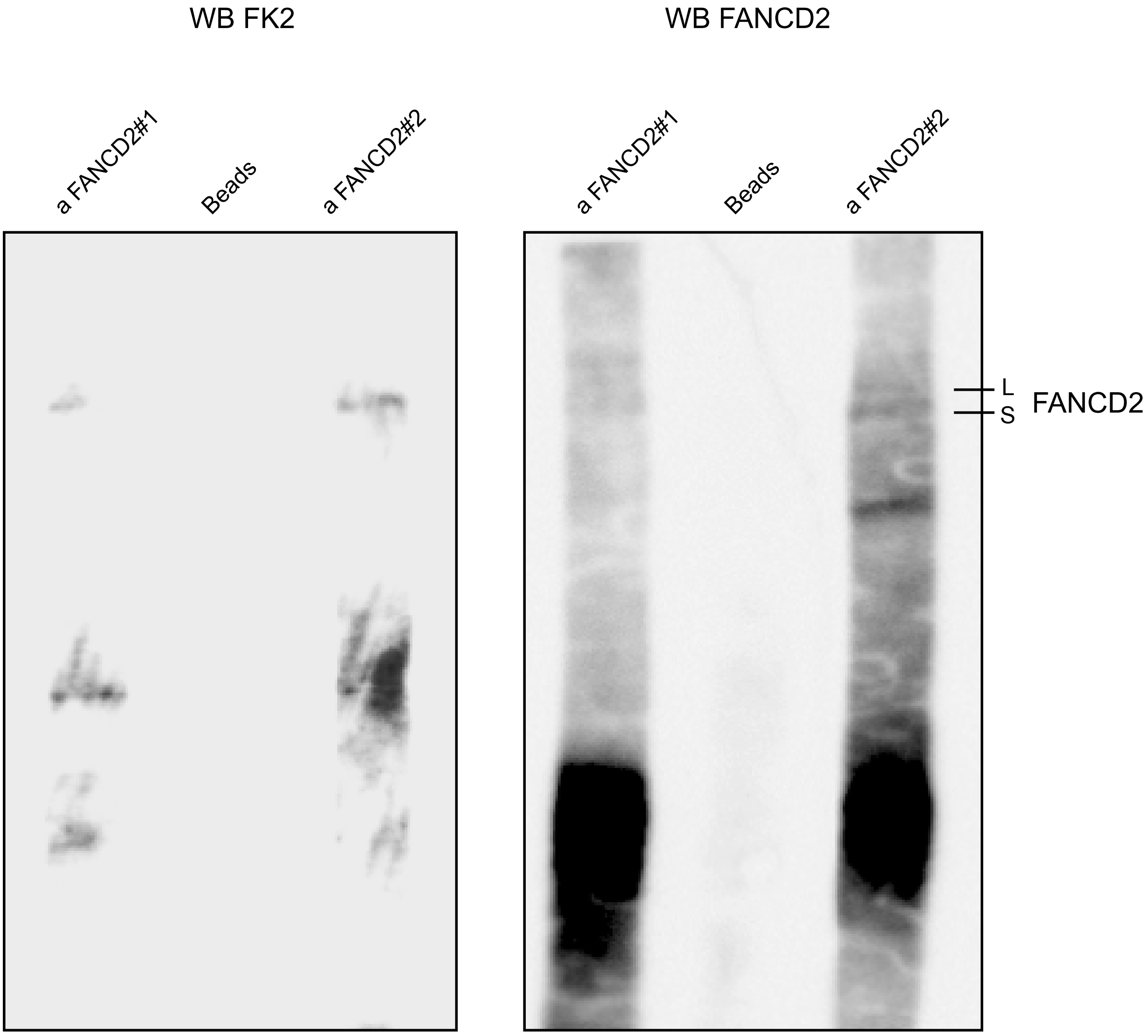


**Fig. S6** Western blot with mono- and polyubiquitinylated conjugates (FK2) and FANCD2 antibodies after FANCD2 immunoprecipitation with two different FANCD2 antibodies

**Bezine et al., 2016**

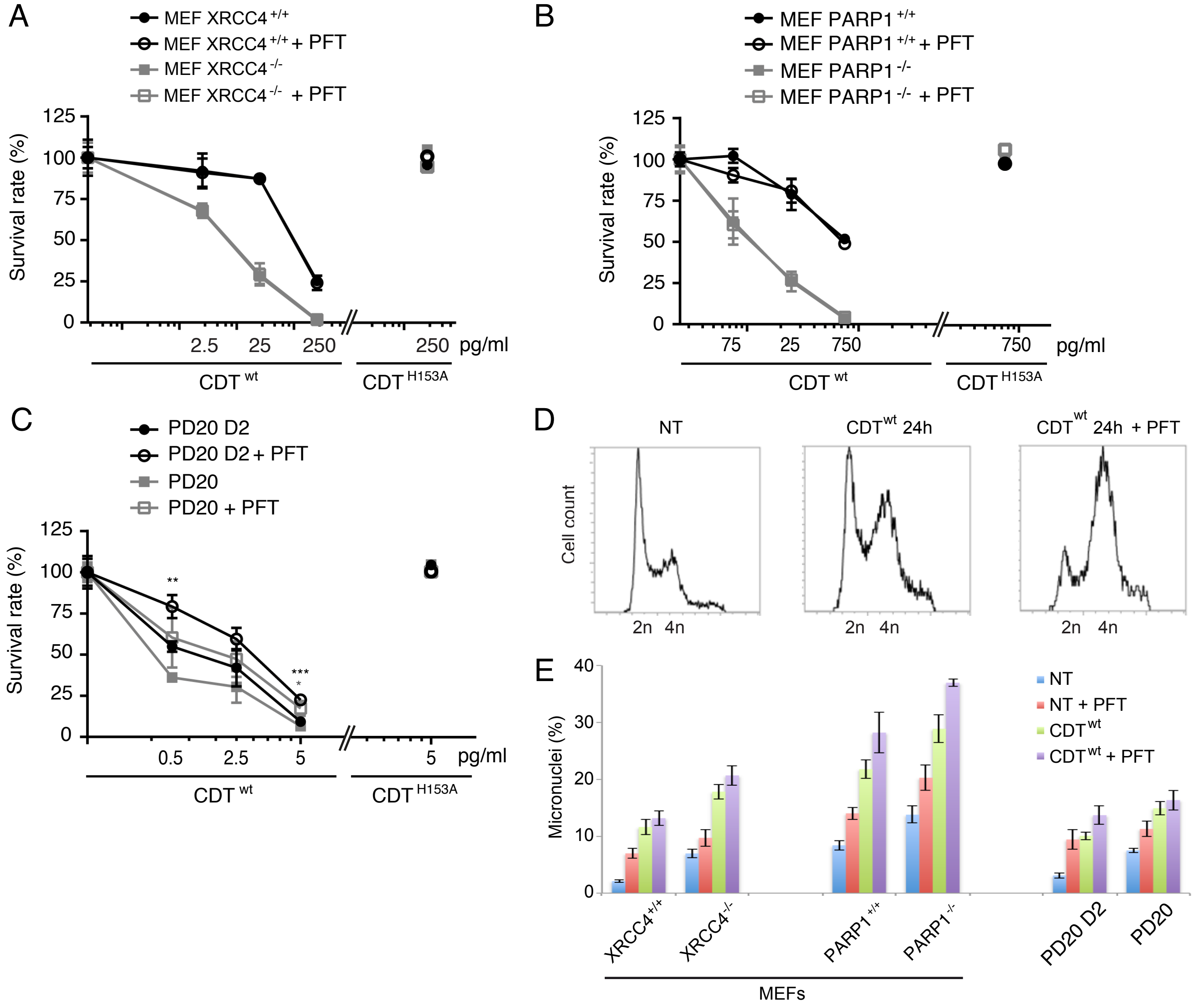


**Fig. S7** Effect of p53 inhibition on cell survival of MEFs and PD20 cells exposed to CDT

**Bezine et al., 2016**

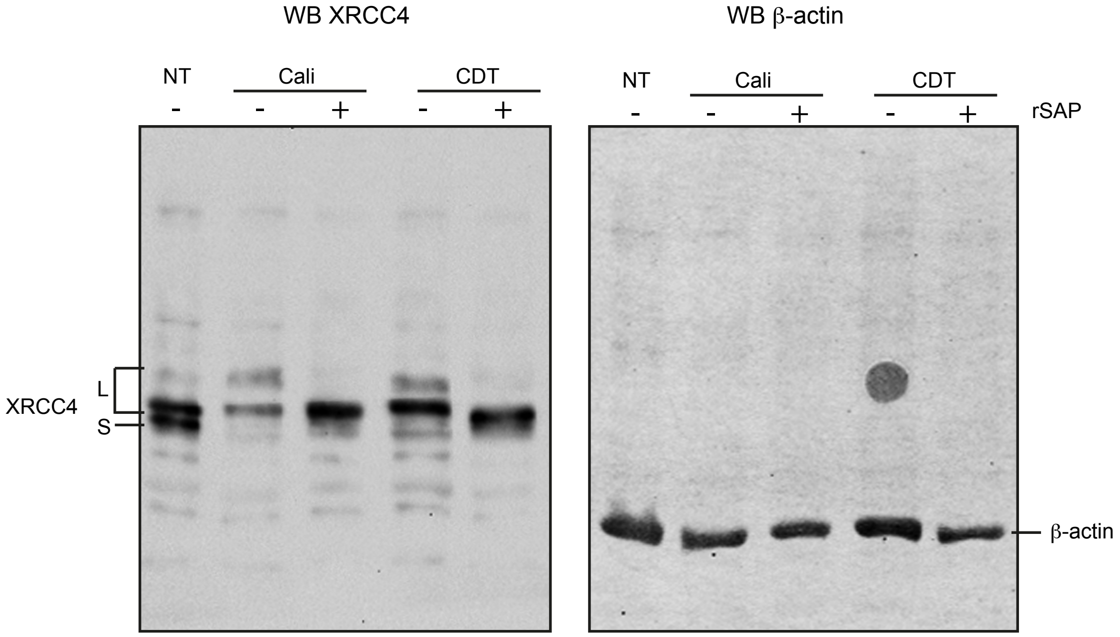


**Fig S8.** Full-lenght blots from Fig. 2B

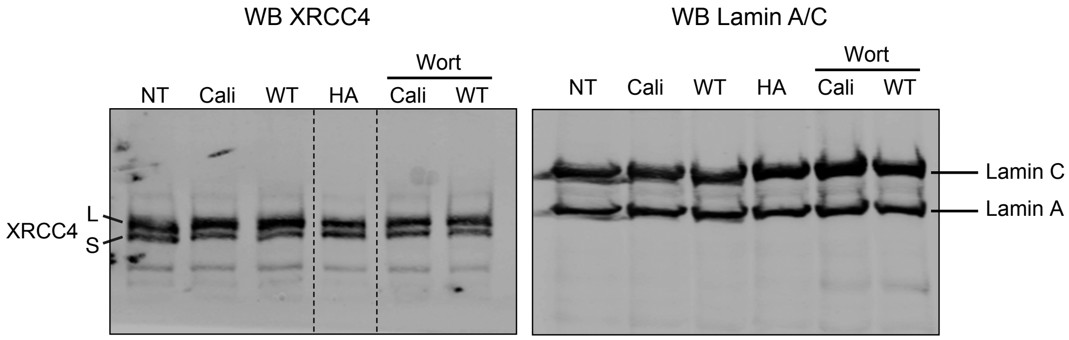


**Fig S8.** Full-lenght blots from Fig. S3

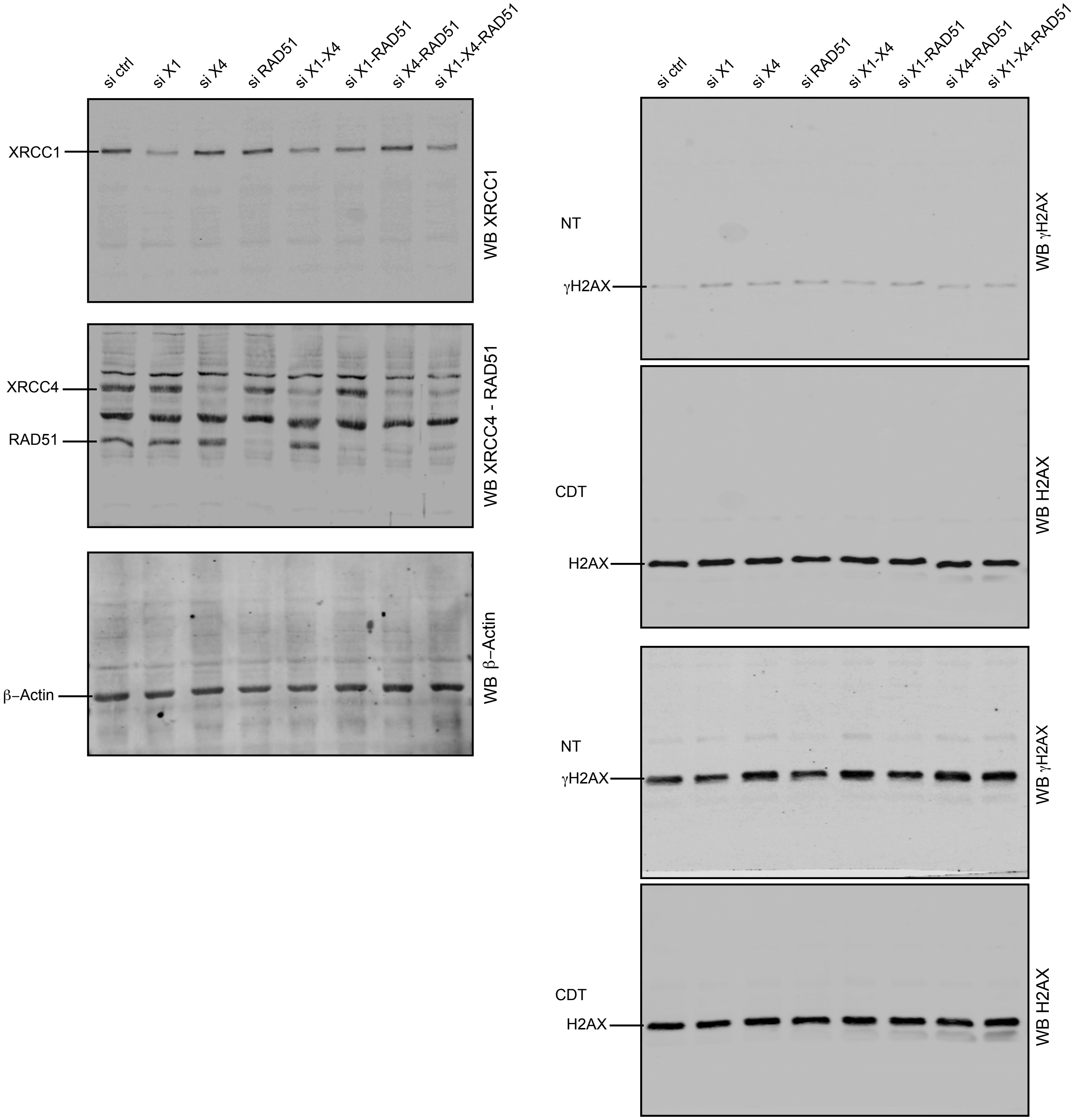


**Fig S8.** Full-lenght blots from Fig. 4A and 4C

**Bezine et al., 2016**

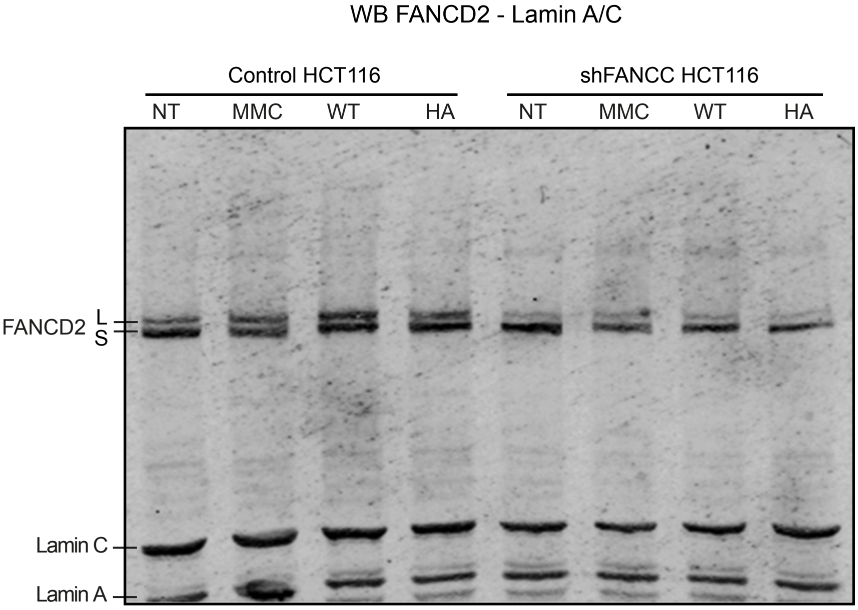


**Fig S8.** Full-lenght blots from Fig. 5B

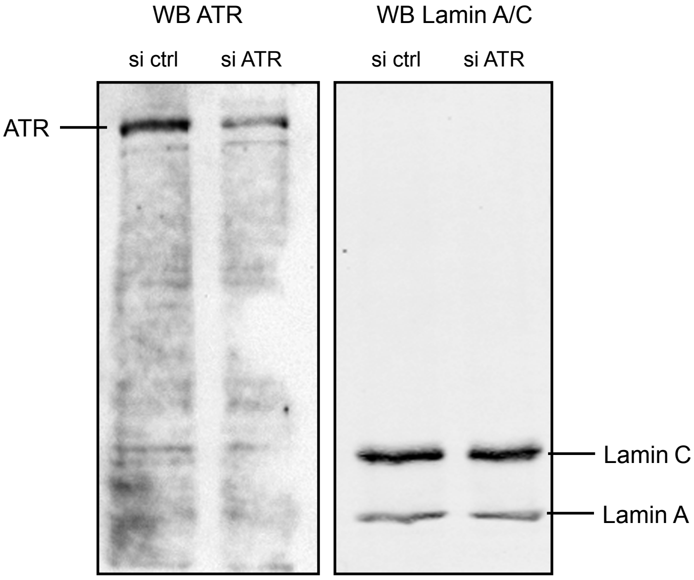


**Fig S8.** Full-lenght blots from Fig. 6A

**Bezine et al., 2016**

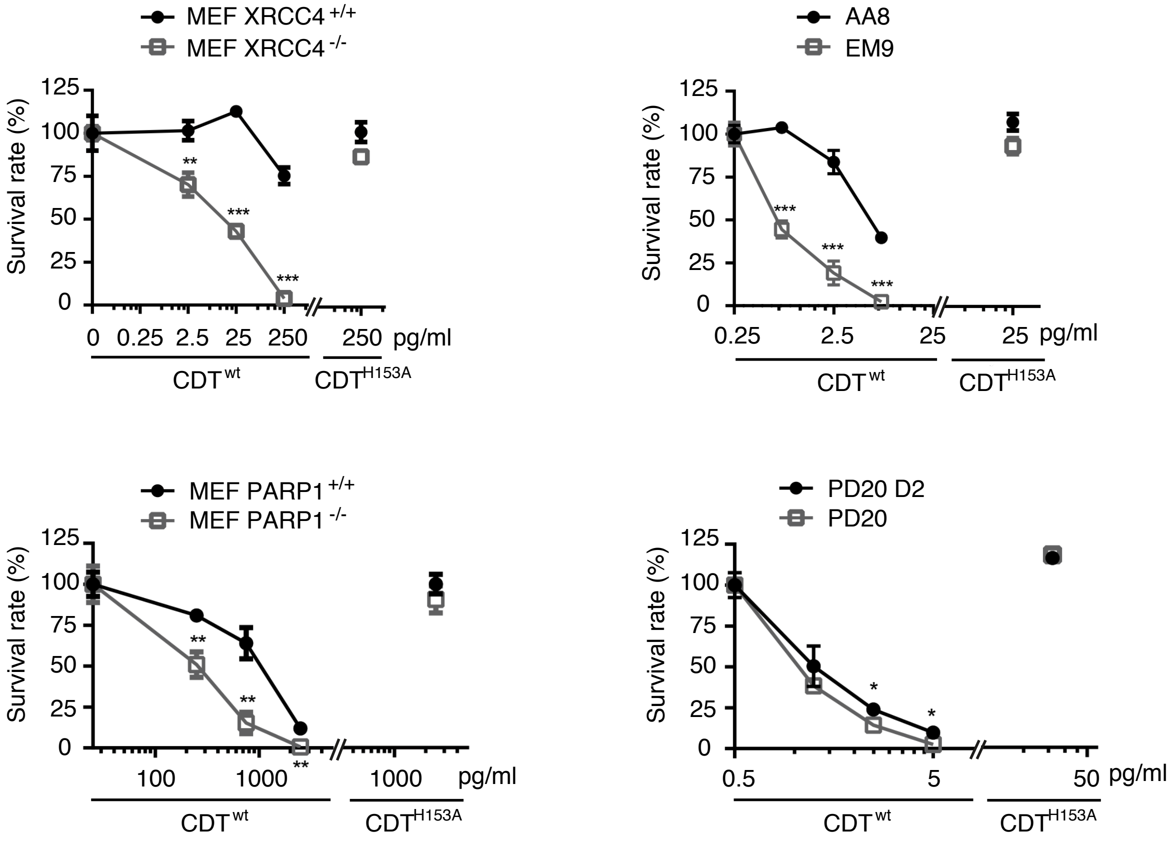


**Fig. S9** Representation in linear scale of the quantification of clonogenic assays presented in Fig. 2A, 3A-B and 5A

**Bezine et al., 2016**
